# Promoting Effect of Horticultural Therapy on College Students’ Positive Psychological Quality

**DOI:** 10.3389/fpsyg.2022.864147

**Published:** 2022-04-26

**Authors:** Yong-ling Li, Feng Li, Zhi Gui, Wen-bin Gao

**Affiliations:** ^1^Department of Psychology, Institute of Psychology, Chinese Academy of Sciences, Beijing, China; ^2^Department of Psychology, Chinese Academy of Sciences, Beijing, China; ^3^Students’ Affairs Division, Tianjin Agricultural University, Tianjin, China; ^4^College of Agriculture and Resources and Environment, Tianjin Agricultural University, Tianjin, China

**Keywords:** horticulture therapy, positive psychological quality, emotional dimension, transcendental dimension, college students

## Abstract

To explore the effect of horticultural therapy on cultivating College Students’ positive psychological quality and to provide reference for college students’ mental health education, 176 college students were randomly divided into experimental group (*n* = 88) and control group (*n* = 88). The experimental group was intervened by horticulture therapy, and the *Chinese college students’ mental health evaluation system* and *Chinese college students’ positive psychological quality scale* were used to test the experimental group and the control group. There was no difference in the six dimensions of positive psychological quality between the experimental group and the control group in the pre-test. After 9 weeks of intervention, the differences between the experimental group and the control group in the “cognitive dimension” and “emotional dimension” were significant. The scores of emotional dimension and transcendental dimension in the experimental group were higher than those in the pre-test (all *p* < 0.05); meanwhile, there is no difference in the other four dimensions. Horticultural therapy can promote the positive psychological quality of college students.

## Introduction

Horticulture Therapy (HT) is a horticultural activity for the purpose of healing. Through the guidance of professionals, it provides people with the opportunity to get in close contact with plants, promote the development of personal thoughts and emotions, and live in harmony with the environment (Relf, 1992). The healing effect of garden and nature can be traced back to ancient Greece and modern times (Shoemaker, 2019). HT is the practice of using horticultural activities to treat and rehabilitate human beings. Its core is to maximize the improvement of human physiology/psychology, cognition, society, and health (Haller and Capra, 2006). It is called horticultural welfare in Taiwan, HT in Japan, and horticultural therapy in South Korea (Xiao, 2015). The definition given by American Horticulture Therapy Association (AHTA) is an effective method to adjust and update people who need to improve their physical and mental aspects from social, educational, psychological, and physical aspects by using plant cultivation and horticultural operation activities (Gonzalez and Kirkevold, 2014). Chinese scholar Li Shuhua put forward the concept of HT (Li, 2000a,b). Then, it is defined as: HT refers to an effective method to maintain and restore people’s physical and mental functions and improve the quality of life through plants, plant growth environment, and various activities related to plants (Li, 2011).

## Development and Research Status of HT

### Development of HT

The use of gardening to calm the senses dates back to Mesopotamia in 2000 BC. Around 500 BC, Persians began to build gardens to delight their senses by combining beauty, fragrance, music (running water), and coolness (Shoemaker, 2019). Since the 19th century, people in the United States have begun to understand the therapeutic effect of quiet garden environment (Detweiler et al., 2012). Dr. Benjamin Rush, who is regarded as the “Father of American Psychiatry” in the United States, talked about the efficacy of garden environment for psychiatric patients in his book medical investigation and observation of mental diseases (Davis, 1997). The rehabilitation of wounded veterans after World War I contributed to the application of horticulture in clinical environment (Lewis, 1976). After World War II, vocational training was gradually introduced into horticulture. At present, horticultural therapists participate in education and training institutions serving special groups, such as hospitals, rehabilitation centers, nursing homes, and schools for the disabled in some countries (Relf, 1992; Du, 2014; Fu, 2016).

### Research Status of HT

Nicholas found that HT significantly improved the quality of life, anxiety, depression, and social relations, physical, and cognitive effects of the elderly (Nicholas, 2019). Xu (2019) found that foreign studies at the end of the 19th century showed that students’ mental health level and literacy were significantly improved in gardening related activities. In their research, Beech et al., Shannon et al., and Stigsdotter and Graham in the United States found that letting students complete some campus gardening activities will deepen students’ feelings of nature.

In China, HT is in the initial stage of development due to its late start, and there is a certain gap with other countries in the richness of practical experience and theoretical knowledge (Gonzalez and Kirkevold, 2014). According to the statistics of China National Knowledge Infrastructure (CNKI), it was not until 2000 that Li Shuhua systematically summarized and summarized the concept and efficacy of HT for the first time.

Firstly, CNKI database is used as the data source for advanced retrieval of journal literature. The retrieval conditions are “HT” and “college students,” and the retrieval form is “subject.” The time span is 2000–2020. A 33 retrieval results are obtained. Through visual analysis, the following data are obtained.

**Figure 1:**
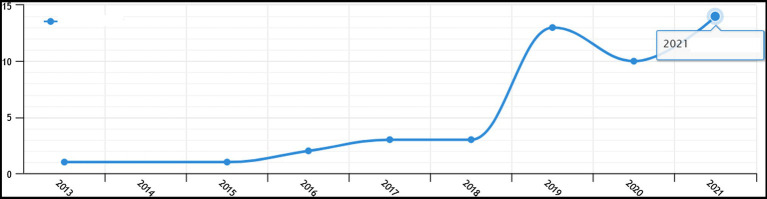
China National Knowledge Infrastructure (CNKI) based analysis of the overall trend of HT and college students’ research literature in China from 2000 to 2020.

Figure 1 shows the publication status of HT and college students’ research literature in China from 2000 to 2020 based on CNKI. In terms of quantity, the research on HT and college students was not published until 2013, and then, the development was slow. The 2018 period is a period of rapid growth, during which the number of papers gradually increases. The analysis predicts that the number will reach a new high in 2021. From the perspective of theme analysis, the top five are HT, mental health, college students’ mental health education, mental health education, and positive psychological quality. In recent years, some scholars have conducted a series of studies on HT and discussed the role of HT in college students’ mental health education.

Most of the studies on the construction of HT and its implementation in college students’ mental health education in China from a theoretical perspective began after 2010. Wu and Qiu (2013) summarized the operation steps and psychological experience of HT based on the experiment of HT and proved that HT can promote college students to love nature and life, understand the meaning of life through labor, and establish a positive outlook on life. Cai and Li (2015) analyzed the efficacy of HT in spiritual, social, and physical aspects, as well as the unique advantages of the Academy of Agricultural Sciences in developing HT. And they believed that the promotion and popularization of HT in the traditional mental health education and the development of HT practice activities will help to temper the will of college students and cultivate their positive sunshine mentality. Liang and Shi (2018) studied the connotation and evolution of HT, the theoretical basis, the role of HT in cultivating college students’ mental health, and its implementation strategies, and believed that HT is a combination of agronomy, behavior, art, psychology, and other disciplines to help students achieve a happy and exciting life state with the help of fresh plants. Song (2017) analyzed the necessity and feasibility of HT in college students’ mental health education and discussed the application and curative effect of HT in campus. Zhang et al. (2017) analyzed the advantages of students majoring in traditional Chinese medicine in agricultural colleges in HT-related work. Yang and Ding (2019) applied HT to the practice of mental health education for college students at colleges and universities of traditional Chinese medicine. Zhao et al. (2020) explored the strategy of effectively promoting HT among college students through questionnaire analysis.

In terms of intervention research on the mental health of college students with HT, the research results of Liu (2018), Qi et al. (2019), and Gao et al. (2019) show that HT has a positive therapeutic effect on the mental health of college students. HT can enhance college students’ self-awareness and psychological adjustment ability and experience the significance of “team” and “process.” It can not only expand the perspective and depth of HT, but also provide effective auxiliary means for mental health education at colleges and universities. However, on the whole, the research and practice of HT on university campus in China are still in its infancy, and there are few cases for reference.

In 2018, the “Guiding Outline of Mental Health Education for College Students” issued by the Ministry of Education pointed out that it is necessary to strengthen the popularization and dissemination of mental health knowledge, fully tap students’ psychological potential, cultivate positive psychological quality, and promote the harmonious development of students’ body and mind (Party group of the Ministry of education of the Communist Party of China, 2018). Because positive psychological quality has an important value orientation in mental health education, this study attempts to help students overcome negative emotions and strengthen their pressure resistance through novel and interesting experience activities, so as to promote students to show a positive spirit (Li, 2020).

In order to understand the intervention effect of HT, six dimensions of positive psychological quality were selected to evaluate the intervention effect. This not only helps to establish appropriate training methods of positive psychological quality, but also provides effective theoretical support and practical basis for students’ mental health intervention system.

## Research Objects and Methods

### Research Objects

This study uses the College Students’ Mental Health Screening Scale in the Chinese college students’ mental health evaluation system to screen the students with psychological problems such as somatization, anxiety, and depression and in urgent need of psychological intervention according to the psychological evaluation results of freshmen in a university in Tianjin. They were randomly divided into experimental group and control group, with 88 people in each group.

Before the intervention, the researcher signed the Informed Consent Form with all the students. During the intervention period, the students in the control group did not accept any one or more forms of intervention but only received pre-test and post-test.

### Research Methods

The theoretical basis of HT intervention is five senses stimulation, creating an environment that can stimulate people’s five senses, such as vision, hearing, smell, touch, and taste, so that people can relax their body and mind and achieve the effect of treatment (Zhang et al., 2016). Around 88 people in the experimental group were randomly divided into three groups to receive intervention activities. The intervention activities were divided into nine times. Each time, each group was divided into five to six people as a group. See Table 1 for specific intervention activities. Each intervention lasted 150 min, including warm-up (20 min), gardening (120 min), and feeling sharing (10 min). The warm-up part includes the review of the last activity to promote the students to enter the activity state as soon as possible; the specific intervention activities are progressive from easy to difficult; and in the sharing link, teachers encourage students to talk about their feelings from the perceptual level, analyze them from the rational level, encourage students to pay attention to their emotions and thinking, and actively affirm students’ innovation in experience activities. At the end, students fill in the “Activity Evaluation Questionnaire.”

**Table 1:** HT activity plan.

No.	Name	Content	Target
1	Walking hand in hand	Team building, contract making, floral meditation.	Understand HT and experience teamwork and interpersonal interaction.
2	Color decompression	Explain the color theory and guide students to practice the emotional expression between color and painting.	Feel the expression of emotion through works of art.
3	Cultivation and planting	Explain cultivation and planting techniques, and guide students to use seedling hole plate to plant cucumber, colored pepper and other plants on site.	Experience the magic and greatness of life and the touch of soul.
4	Gardening	Raw materials such as vegetables and fruits are processed into stacked paintings.	Present the love of life, the thinking of life and the feeling of life.
5	Micro landscape production	Plants with similar growth environment such as moss and meat are combined with various landscape dolls, and potted plants are planted by using the principle of aesthetic composition.	The presentation of rich personal inner world.
6	Embossing process	Demonstrate the pressing process of dried flowers and guide students to press dried flowers by themselves.	Master the dry flower pressing technology and experience the sense of achievement.
7	Embossed key chain	Demonstrate the production process of embossed key chain and guide students to make it.	Master the production technology of embossed key chain to obtain a sense of achievement and spiritual touch.
8	Walk around the campus	Share the pictures of previous trips in groups and go out to watch the gardening landscape on campus.	Improve the thinking path: expand ideas, experience achievements, and obtain hope and satisfaction.
9	Work display	Display of individual works and selection of excellent works.	Experience the sense of achievement, obtain the sense of satisfaction, and condense the team spirit.

### Tools

#### Mental Health Evaluation System for Chinese College Students

The Mental Health Evaluation System for Chinese College Students is a mental health evaluation system compiled by the Ministry of Education according to the age, psychological characteristics and social conditions of college students, and the psychological characteristics of college students. This study selected Chinese college students’ Mental Health Scale (CCSMHS) compiled by Zheng Richang of Beijing Normal University to measure college students’ psychological distress symptoms and reflect the damage to their mental health, which was compiled in 2005. In the researcher’s report, the internal consistency reliability of each dimension of the scale is between 0.772 and 0.893 and the test–retest reliability is between 0.352 and 0.804; each dimension of the scale has different degrees of positive correlation with each dimension of SCL-90, most of which are significant, and there is a very significant negative correlation with the total scale and subscale of Chinese college students’ adaptation; the consistency percentage between the results of each dimension of the scale and the expert diagnosis results was more than 60%, and the average consistency percentage was 78.62%; and confirmatory factor analysis was used for the scale. The NFI, NNFI, RFI, and CFI values of each subscale were greater than 0.90, and most of the RMSEA values were less than 0.1, indicating that the scale has good reliability and validity and is suitable for this study (Zheng et al., 2005). The scale consists of 12 dimensions: somatization, depression, anxiety, compulsion, paranoia, psychotic tendency, inferiority complex, sexual psychological disorder, dependence, impulse, withdrawal, and aggression. It can be used to find students who may need special help because of their psychological symptoms (Shen and Liang, 2006).

#### Positive Psychological Quality-Related Scale

In order to understand the intervention effect of HT on College Students’ Positive Psychological Quality, the Chinese college students’ positive psychological quality scale was used to evaluate (Meng and Guan, 2009). The scale was developed by Meng Wanjin and Guan Qun based on large sample measurement and factor analysis. It contains 62 questions and has good reliability and validity. Table 2 lists in detail the six dimensions and 20 sub-items in the positive psychological quality table of Chinese college students. This study will investigate the intervention effect before and after HT from the above six dimensions. The scale adopts Likert 5-point positive scoring method, “1” means very inconsistent and “5” means very consistent. The higher the score, the better the psychological state.

**Table 2:** Dimensions and contents of positive psychological quality of Chinese college students.

Dimension	Contents	
Cognition	Creativity, curiosity, love of learning, thinking ability	Interpersonal relationship
Sincere, brave, persistent, and enthusiastic	Emotions	Feel love and kindness, social wisdom
Justice	Team spirit, integrity and fairness, and leadership	Self-control
Tolerance, modesty, prudence, and self-control	Surpass	Soul touch, hope and faith, and humor

This study investigated the effects of HT intervention from six dimensions: cognition and interpersonal. The internal consistency reliability of the questionnaire is 0.652–0.922, indicating that the scale has good reliability.

### Statistical Method

The pre-test and post-test data are preliminarily entered and sorted out by Excel and SPSS 20 The dimensions of college students’ positive psychological quality questionnaire were statistically analyzed. Count the number of data cases (percentage), and *χ^2^* test is used for comparison between groups. The measurement data conforming to the normal distribution are expressed in mean ± SD, and the independent sample *t*-test is used for the comparison between groups; the non-normal distribution is represented by the median (maximum and minimum), and the *t*-test is used for the comparison between groups. Paired sample *t*-test was used to compare the measurement data of the same group before and after intervention.

## Research Results

### Composition of Experimental Group and Control Group

The demographic statistical variables of the tested students are shown in Table 3. It can be seen from Table 3 that there is no difference in personnel composition between the experimental group and the control group; there was no significant difference between boys and girls in the experimental group and the control group; and Chi square test was used to test the proportion difference between urban students and rural students in the experimental group and the control group, and the results were also not significant. This shows that there is no difference in personnel composition between the experimental group and the control group, which is random and meets the requirements of equal histochemical grouping of this study.

**Table 3:** Basic composition and difference test of experimental group and control group.

	Male		Female		City		Village	
	*n*	%	*n*	%	*n*	%	*n*	%
Experimental group	38	43.182	50	56.818	41	46.591	47	53.409
Control group	40	45.455	48	54.545	42	47.727	46	52.273
χ^2^	0.092				0.023			
*p*	0.762				0.880			

### Comparison of Pre-test Differences Between Experimental Group and Control Group

The pre-test differences of the tested students are shown in Table 4. It can be seen from Table 4 that before the intervention, there was no difference in the six dimensions of positive psychological quality between the experimental group and the control group, which was random and met the requirements of equal histochemical grouping of this study.

**Table 4:** Comparison of pre-test differences between experimental group and control group.

	Experimental group (88 people)		Control group (88 people)		*t*	*p*
	*M*	*SD*	*M*	*SD*		
Cognition	27.602	6.514	28.341	6.055	−0.779	0.437
Interpersonal relationship	24.159	5.677	24.523	5.575	−0.429	0.669
Emotions	26.557	6.500	27.307	5.939	−0.799	0.425
Justice	21.205	5.496	21.989	5.134	−0.978	0.329
Self-control	23.578	5.969	24.250	5.086	−0.802	0.424
Surpass	23.625	6.143	23.307	5.826	0.353	0.725

### Comparison of Pre-test and Post-test Differences Between Experimental Group and Control Group

The pre-test and post-test results of the experimental group and the control group in six dimensions are shown in Table 5. As can be seen from Table 5, compared with the control group, the difference in “emotions” and “surpass” of the experimental group has reached a significant level; other test items did not reach a significant level. This shows that the nine intervention activities not only increase the interaction between students, teachers, and students, but also cultivate interpersonal skills. At the same time, it also arouses students’ inner creativity and artistic inspiration to heal psychological trauma. By contacting the expression of various plants and artistic creativity, it specifically expresses their inner emotions or ideas on plants and works, helps students recognize the existence of these emotions and ideas, and greatly improves their cognition of self-worth. Finally, the emotional dimension and transcendence dimension of the tested students show an improvement effect.

**Table 5:** Comparison of pre-test and post-test differences between the experimental group and the control group.

		Levene test with equal variance *T*-test of equal mean				
		F test	Significance	t	Freedom	Significance (two tailed)
Cognition	Assuming equal variance	0.916	0.34	0.431	174	0.667
	The variance is not assumed to be equal			0.446	173.874	0.657
					
Interpersonal relationship	Assuming equal variance	1.735	0.19	0.575	174	0.566
	The variance is not assumed to be equal			0.629	172.224	0.531
					
Emotions	Assuming equal variance	0.172	0.679	2.013	174	**0.046**
	The variance is not assumed to be equal			2.004	173.411	**0.048**
					
Justice	Assuming equal variance	0.927	0.338	0.119	174	0.906
	The variance is not assumed to be equal			0.125	173.647	0.901
					
Self-control	Assuming equal variance	0.013	0.91	0.713	174	0.477
	The variance is not assumed to be equal			0.720	169.379	0.473
					
Surpass	Assuming equal variance	1.18	0.28	2.386	174	**0.019**
	The variance is not assumed to be equal			2.280	173.626	**0.025**

Bold values indicate significant differences between the data.

## Conclusion and Countermeasure Research

### Promoting Effect of HT on College Students’ Positive Psychological Quality

This study shows that HT has a certain effect on improving college students’ psychological status and positive psychological quality, especially on students’ “surpass” and “emotions.” This conclusion is similar to that of Zhang (2014).

The students who did not participate in HT still followed their previous communication methods and communication paths and did not change. The students in the experimental group concretely express their inner emotions or ideas on plants and works by contacting various plants and the expression of artistic creativity. This not only helps students recognize the existence of these emotions and ideas, but also relieves students’ existing negative emotions such as anxiety and depression, enhances self-recognition, increases the interaction between the tested students, teachers, and students, cultivates interpersonal skills, and arouses students’ inner creativity and artistic inspiration. This can cure psychological trauma, alleviate emotional conflict, emotional depression or anxiety, and finally make students’ emotional dimension and transcendence dimension show an improvement effect, which is significantly different from the control group. This conclusion is similar to that of Wang (2019) and Zhou (2018a).

### Places for Improvement in This Research

Firstly, it should be further broadened from the aspects of seasonal arrangement, richness of forms, and selection of materials.

There were nine intervention activities, including stacking paintings, micro landscape production, and embossing crafts production; the course was held from September to November 2018, in autumn and winter. Due to the impact of the natural environment, only indoor experience activities can be carried out. In the future, we will try to adjust the course time to spring and summer, add outdoor cultivation and planting activities, and let students fully experience the whole process of life cycle. To cultivate the richness of activity forms, we should design corresponding activity contents and environment based on people’s five senses: vision, hearing, smell, touch, and taste, perceive things through senses, understand the environment, carry out plant cultivation and planting activities, and experience the life cycle of plants. (Li, 2018a) In addition, the university has relatively abundant spare time. In the future, elective courses related to HT can be opened, such as plant cultivation, flower arrangement and embossing, and students can be organized to experience and experiment as much as possible, so as to deepen the teaching effect Song (2017).

Secondly, we can carry out interdisciplinary activities to improve the professionalism of experience activities. At present, most of the teachers leading HT experience activities in our school are teachers engaged in psychological education, so the knowledge of the course is slightly insufficient. Li Shuhua believes that a scientific and effective HT evaluation system should be established in HT, interdisciplinary, and comprehensive, research methods and research contents should be diversified, and HT should be developed into a mature adjuvant therapy system Li (2018b) Therefore, we can try to hire teachers of different majors to participate in HT experience activities in the future. For example, hire teachers majoring in art to teach students how to sketch plants, ask teachers majoring in biology to explain plant photosynthesis, and ask teachers majoring in plant protection to teach students how to prevent and control diseases and pests. Plants from sowing seeds to germination, growth to decline, and death are the same process as people’s life process. By being close to plants and mobilizing people’s five sensory systems of vision, smell, hearing, touch, and taste, we can help people build self-confidence and obtain a sense of achievement, so as to achieve the purpose of physical and mental health Zhou (2018b).

## Conclusion

At present, the theoretical research and specific measures of using HT to improve people’s physical and mental health are still in the initial stage, especially the research and intervention on cultivating college students’ positive psychological quality is still a blank field. This topic will explore the horticultural treatment methods and operation processes suitable for college students from a new perspective, explore the theory and practice of Chinese college students’ psychological intervention system, provide new ideas for college mental health education, and provide reference for the establishment of social psychological service system.

This study examines the intervention effect of HT on college students’ positive psychological quality from a quantitative perspective. Based on this study, future research can try to use the form of case study to more deeply and carefully present the research process of HT and explore the mechanism of the experimental results. Other methods can also be used to study HT, giving HT more diversified research approaches.

## Data Availability Statement

The original contributions presented in the study are included in the article/supplementary material; further inquiries can be directed to the corresponding author.

## Author Contributions

Yl-L contributed to conception and design of the study and wrote the first draft of the manuscript. FL performed the statistical analysis. ZG and W-bG wrote the sections of the manuscript. All authors contributed to the article and approved the submitted version.

## Funding

This work was supported by Tianjin Students’ Mental Health Education Development Center Project (XLZX-G201802).

## Conflict of Interest

The authors declare that the research was conducted in the absence of any commercial or financial relationships that could be construed as a potential conflict of interest.

## Publisher’s Note

All claims expressed in this article are solely those of the authors and do not necessarily represent those of their affiliated organizations, or those of the publisher, the editors and the reviewers. Any product that may be evaluated in this article, or claim that may be made by its manufacturer, is not guaranteed or endorsed by the publisher.
